# A seven-membered cell wall related transglycosylase gene family in *Aspergillus niger* is relevant for cell wall integrity in cell wall mutants with reduced α-glucan or galactomannan

**DOI:** 10.1016/j.tcsw.2020.100039

**Published:** 2020-03-21

**Authors:** Tim M. van Leeuwe, Jasper Wattjes, Anna Niehues, Gabriel Forn-Cuní, Nicholas Geoffrion, Hugo Mélida, Mark Arentshorst, Antonio Molina, Adrian Tsang, Annemarie H. Meijer, Bruno M. Moerschbacher, Peter J. Punt, Arthur F.J. Ram

**Affiliations:** a Leiden University, Institute of Biology Leiden, Molecular Microbiology and Biotechnology, Sylviusweg 72, 2333 BE Leiden, the Netherlands; b Institute for Biology and Biotechnology of Plants, University of Muenster, Schlossplatz 8, 48143 Münster, Germany; c Leiden University, Institute of Biology Leiden, Animal Science and Health, Einsteinweg 55, 2333CC Leiden, the Netherlands; d Centre for Structural and Functional Genomics, Concordia University, Quebec H4B1R6, Canada; e Centro de Biotecnología y Genómica de Plantas, Universidad Politécnica de Madrid (UPM)-Instituto Nacional de Investigación y Tecnología Agraria y Alimentaria (INIA), Campus Montegancedo-UPM, 28223 Pozuelo de Alarcón (Madrid), Spain; f Departamento de Biotecnología-Biología Vegetal, Escuela Técnica Superior de Ingeniería Agronómica, Alimentaria y de Biosistemas, Universidad Politécnica de Madrid, 28040 Madrid, Spain; g Dutch DNA Biotech, Hugo R Kruytgebouw 4-Noord, Padualaan 8, 3584 CH Utrecht, the Netherlands

**Keywords:** Chitin, Crh transglycosylases, α-glucan, Cell wall integrity, *Aspergillus niger*

## Abstract

Chitin is an important fungal cell wall component that is cross-linked to β-glucan for structural integrity. Acquisition of chitin to glucan cross-links has previously been shown to be performed by transglycosylation enzymes in *Saccharomyces cerevisiae*, called Congo Red hypersensitive (Crh) enzymes. Here, we characterized the impact of deleting all seven members of the *crh* gene family (*crhA-G*) in *Aspergillus niger* on cell wall integrity, cell wall composition and genome-wide gene expression. In this study, we show that the seven-fold *crh* knockout strain shows slightly compact growth on plates, but no increased sensitivity to cell wall perturbing compounds. Additionally, we found that the cell wall composition of this knockout strain was virtually identical to that of the wild type. In congruence with these data, genome-wide expression analysis revealed very limited changes in gene expression and no signs of activation of the cell wall integrity response pathway. However, deleting the entire *crh* gene family in cell wall mutants that are deficient in either galactofuranose or α-glucan, mainly α-1,3-glucan, resulted in a synthetic growth defect and an increased sensitivity towards Congo Red compared to the parental strains, respectively. Altogether, these results indicate that loss of the *crh* gene family in *A. niger* does not trigger the cell wall integrity response, but does play an important role in ensuring cell wall integrity in mutant strains with reduced galactofuranose or α-glucan.

## Highlights

• *Aspergillus niger* has seven chitin transglycosylation (*crh*) gene orthologues.
• All seven *crh* genes (*crhA-G*) are dispensable for growth and development.
• Disruption of *crhA*-*G* neither weakens the cell wall nor triggers the CWI response.
• Crh enzymes are relevant for CWI in absence of either α-glucan or galactofuranose.
• α-Glucan compensates for Crh loss in chitin-to-cell-wall tethering.

## 1 Introduction

The fungal cell wall is a rigid, yet dynamic structure that dictates cell shape, provides protection from other microbes, helps to colonize new environments and is required for virulence in pathogenic fungi. The fungal cell wall consists of different polymeric sugars, including α-1,3-glucans, mixed α-1,3/1–4-glucans, β-1,3-glucans, β-1,6-glucans and mixed β-1,3/1,4 or β-1,3/1,6 glucan varieties, chitin (β-1,4-*N*-acetyl glucosamine polymer), galactomannan and glycoproteins (Free, 2013, Gow et al., 2017, Ruiz-Herrera and Ortiz-Castellanos, 2019). In filamentous fungi, β-1,3-glucans and chitin comprise the majority of these polymers, forming intrachain hydrogen bonds that can assemble into microfibrils to form a scaffold around the cell (Gow et al., 2017). Additionally, β-1,3-glucans act as a backbone structure wherefrom branching, through β-1,6-glucan linkages that constitute 3% to 4% of the total glucan linkages (Latgé, 2007), creates multiple β-1,3-glucan non-reducing end acceptor sites. These non-reducing ends can be used for covalent cross-links for other β-1,3-glucans, chitin and mannan, facilitated by Gel/Gas/Php, Crh and Ktr/Mnt/Mnn families, respectively (Cabib et al., 2008, Cabib et al., 2007, Fontaine et al., 2000, Fonzi, 1999, Hartland et al., 1996, Henry et al., 2016, Mouyna et al., 2000, Rodríguez-Peña et al., 2000, Samalova et al., 2017).

Prior to cross-linking, both β-1,3-glucan and chitin are synthesized at the plasma membrane by β-glucan and chitin synthases using UDP-glucose and UDP-*N*-acetyl-glucosamine, respectively. Polymers are extruded into the periplasmic space, where chitin becomes cross-linked to glucan by GPI-anchored Crh transglycosylases (Arroyo et al., 2016). Crh enzymes exhibit both chitinase and transglycosylation activity, effectively hydrolyzing a chitin donor molecule followed by subsequent (re-)attachment to acceptor substrates. These acceptor substrates are either chitin (homotransglycosylation) or β-1,3 and β-1,6-glucans (heterotransglycosylation), respectively (Mazáň et al., 2013). Crh enzymes can be classified as part of either of the fungal specific GH16_18/19 subfamilies (Viborg et al., 2019). These chitin to β-1,3-glucan cross-linking enzymes have been most extensively studied in *Saccharomyces cerevisiae* on both functional and structural level (Blanco et al., 2015, Cabib et al., 2008, Cabib et al., 2007, Rodríguez-Peña et al., 2000). Named Congo Red Hypersensitive (CRH), *CRH1* and *CRH2* showed redundant function in the covalent attachment of chitin to glucan, and exhibited sensitivity towards Congo Red (CR) and Calcofluor White (CFW) in both single and double knockouts strains. *CRH1* and *CRH2* were found to be induced during vegetative growth whereas a third, *CRH* related gene (*CRR1*) encoding an additional transglycosylase, was only induced during sporulation, concomitantly with *CRH1.* A single knockout of *CRR1* did not result in hypersensitivity to either CR or CFW (Rodríguez-Peña et al., 2000), but was shown to have a role in formation of the ascospore cell wall (Gómez-Esquer et al., 2004). In *Candida albicans,* single, double and triple knockouts of the three Crh paralogues, *UTR2*, *CRH11* and *CRH12* resulted in sensitivity towards CR, and avirulence in mouse models (Pardini et al., 2006). Furthermore, overexpression of either *CRH1* or *UTR2* in *C. albicans* led to protection against osmotic shock as a result of reduced cell wall elasticity, whereas a triple deletion resulted in increased osmo-sensitivity (Ene et al., 2015).

In contrast to yeast-like fungi, relatively little is known on the function of the Crh enzymes in filamentous fungi. A phylogenetic analysis showed that Crh enzymes are very well conserved in fungal genomes with noticeably more orthologues found in filamentous fungi compared to yeast-like fungi (Arroyo et al., 2016). *Neurospora crassa* and *Aspergillus fumigatus* both encode five orthologues, whereas *A. niger* contains seven orthologues (Pel et al., 2007). All these three fungi displayed wild type growth morphology for all single knockouts strains (Fang et al., 2019, Patel and Free, 2019, van Leeuwe et al., 2019). In case of *A. fumigatus, Af*Crh5p was structurally and functionally characterized as a true transglycosylase with both homo- and heterotransglycosylation activity, additionally identifying both donor and acceptor substrate binding kinetics and moieties (Fang et al., 2019). Unlike the situation in *S. cerevisiae* and *C. albicans,* the quintuple *crh* knockout strain of *A. fumigatus* only showed slight sensitivity towards CR, similar to some of the single knockouts. We previously showed that both single knockouts and seven-fold *crh* gene family knockout in *A. niger* did not display a significant sensitivity towards CR (van Leeuwe et al., 2019). These data indicate that either Crh enzymes play a different physiological role or that *crh* gene loss is compensated by other cell wall components and/or cross-links in filamentous fungi.

A specific difference in cell wall composition between *Aspergilli* and other organisms that have been studied for Crh functionality, *S. cerevisiae* and *C. albicans,* is the presence of α-glucan in filamentous fungi. Recently, the molecular architecture of *A. fumigatus* cell walls was studied in more detail, revealing the importance of α-1,3-glucan for structural integrity (Kang et al., 2018). It was shown that α-1,3-glucan, in addition to the outer cell wall layer, plays an important structural role in the cell wall by forming a rigid hydrophobic core by interaction with chitin. Additionally, they showed that β-glucans have a more mobile and dynamic role in the cell wall, challenging the prevailing paradigm that the chitin-β-glucan matrix provides the load-bearing scaffold of the fungal cell wall (Gow et al., 2017). These findings led us to form a new hypothesis with respect to the function of Crh enzymes in α-glucan containing cell walls of filamentous fungi. In α-glucan containing filamentous fungi, chitin is likely tethered to the cell wall through both β-glucan cross-linking and chitin-α-1,3-glucan hydrophobic interactions, forming a reciprocal and redundant mechanism for chitin-to-cell-wall “anchoring”. As such, removal of all *crh* genes would affect the integrity of the filamentous fungal cell wall to a much lesser extent, than observed in yeasts, which lack α-glucans (Yoshimi et al., 2017).

We previously reported on the construction of a *crh* family knockouts (*crhA-G*) in *A. niger* by seamlessly excising the corresponding ORFs, using CRISPR/Cas9 gene editing (van Leeuwe et al., 2019). Initial phenotypic analysis on cell wall stressing compounds CalcoFluor White (CFW) and CR showed no effects on cell wall integrity. Here, we report on a more extensive analysis of the seven-fold *crh* mutant by analyzing multi-condition gene expression data sets, additional cell stress assays, bioreactor growth, transcriptomic analysis and cell wall composition. Moreover, we analyzed the role of Crh enzymes in the process of cell wall deficiency, by introducing a mutation in a gene required for cell wall galactomannan synthesis (Δ*ugmA*) in the seven-fold *crh* knockout strain. A deletion of *ugmA* was previously shown to compromise cell wall integrity caused by a galactofuranose deficiency (Damveld et al., 2008), and revealed upregulation of *crhE* in transcriptomic studies (Park et al., 2016). Additionally, we assessed the relation between chitin to β-glucan cross-linking and α-1,3-glucan, by reducing cell wall α-glucan synthesis in both wild type and the seven-fold *crh* mutant. We disrupted both the most actively expressed α-glucan synthase E (*agsE*), and cell wall stress induced α-glucan synthase A (*agsA*) (Damveld et al., 2005b). Taken together, our data indicate that Crh enzymes play a significant role in cell wall integrity when galactofuranose is absent and when cell wall α-1,3-glucan levels are reduced.

## 2 Materials and methods

### 2.1 Strains, media, growth conditions

Strains used in this study can be found in Table 1. All media were prepared as described by Arentshorst et al. (2012). In all cases, minimal medium (MM) contained 1% (w/v) glucose, 1.5% agar (Scharlau, Barcelona, Spain) and was not supplemented unless otherwise specified. Complete medium (CM) contained 0.1% (w/v) casamino acids and 0.5% (w/v) yeast extract in addition to MM. Strains were inoculated from −80 °C glycerol stocks onto fresh CM plates and were allowed to grow and sporulate for 5–7 days at 30 °C, prior to spore harvesting. Spores were harvested by addition of 15 mL of 0.9% (w/v) NaCl to CM spore plates and were carefully scraped from the surface with a cotton swab. In case of harvesting spore plates for bioreactor cultivations, 0.05% Tween-80 was added to the 0.9% (w/v) NaCl to prevent spore clumping. Spore solutions were poured over sterile cotton filters (Amplitude™ Ecocloth™ Wipes, Contec Inc., Spartanburg, SC, USA) to remove large mycelial debris. Spore solutions were counted using Bio-Rad TC20™ Automated Cell Counter (Bio-Rad Laboratories, Inc. USA) using Counting Slides, Dual Chamber for Cell Counter (Cat#145-0011, Bio-Rad Laboratories, Inc. USA).

**Table 1:** All strains used in this study.

Name	Genotype	Reference
N402	*cspA1*	Bos et al., 1988
MA234.1	*cspA1, ΔkusA::DR-amdS-DR*	Park et al., 2016
TLF39	*cspA1, ΔkusA::DR-amdS-DR, ΔcrhA-G*	van Leeuwe et al., 2019
MA613.1	*cspA1, ΔkusA::DR-amdS-DR, ΔugmA::hygB*	This study
TLF70	*cspA1, ΔkusA::DR-amdS-DR, ΔcrhA-G, ΔugmA::hygB*	This study
TLF93	*cspA1, ΔkusA::DR-amdS-DR, ΔagsA*	This study
TLF94	*cspA1, ΔkusA::DR-amdS-DR, ΔagsE*	This study
TLF95	*cspA1, ΔkusA::DR-amdS-DR, ΔagsA, ΔagsE*	This study
TLF96	*cspA1, ΔkusA::DR-amdS-DR, ΔcrhA-G, ΔagsA*	This study
TLF97	*cspA1, ΔkusA::DR-amdS-DR, ΔcrhA-G, ΔagsE*	This study
TLF98	*cspA1, ΔkusA::DR-amdS-DR, ΔcrhA-G, ΔagsA, ΔagsE*	This study

### 2.2 Cell wall sensitivity assays

Cell wall disturbing compounds Congo Red (CR), Caspofungin (CA, 0–0.25 µM), sodium dodecyl sulfate (SDS, 0.004% and 0.005%), H_2_O_2_ (0–50 mM) and tunicamycin (0–10 µg/mL) were added to MM plates. Spores were harvested as described above, counted, serially diluted into 2000, 200, 20 and 2 spores/µL and 5 µl of respective dilutions were spotted on MM plates containing cell wall disturbing compounds. Plates were incubated for 3–5 days at 30 °C.

### 2.3 Cell wall isolation and fractionation

#### 2.3.1 Cell wall isolation

Cell wall samples were isolated as previously described (van Leeuwe et al., 2020). In short, dried mycelium was frozen in liquid N_2_ and were ground to break open the cells. Samples were washed to remove intracellular debris and proteins, three times with 1 M NaCl and three times with Milli-Q ultrapure water (MQ). Supernatant was carefully discarded prior to the next washing step. Cell wall samples were lyophilized after washing steps for 48 h.

#### 2.3.2 Cell wall fractionation and dialysis

Lyophilized cell wall samples were weighed using a fine balance (d = ±0.1 mg) using 50 mL plastic tubes. To fractionate cell walls to the first alkali soluble fraction (ASF-I), 1 mL solvent of 80% (v/v) methanol, 5% KOH and 0.1% NaBH_4_ was added per 10 mg cell wall sample. Samples were agitated at 200 rpm at RT for 24 h. Next, samples were spun down for 10 min at 1500 rpm. Harvested supernatant makes up fraction ASF-Ia. A second addition of 1 mL solvent of 80% (v/v) methanol, 5% KOH and 0.1% NaBH_4_ was added to the pellet per original 10 mg cell wall sample, and was incubated at 100 °C for 30 min. Supernatant was harvested after centrifugation (ASF-Ib) and was pooled with ASF-Ia to form the ASF-I fraction. The remaining pellet was taken up in 1 mL solvent of 80% (v/v) methanol, 24% KOH and 0.1% NaBH_4_ per 10 mg cell wall, and repeated in the same way as described above to obtain the ASF-II. The remaining pellet was deemed the alkali insoluble fraction (AIF) and was washed three times with 0.5 M acetic acid, followed by three washes with MQ prior to lyophilization. ASF-I and II supernatants were pH-adjusted (6.0) using glacial acetic acid and freeze-dried after a dialysis step (Spectra/Por MWCO 1000 Daltons, Spectrum Laboratories) against deionized water to remove solutes of a small molecular mass (dialysis tubings were thoroughly washed before use to eliminate any contaminants potentially associated to the membranes) and constituted the alkali-soluble fractions (ASF) I and II respectively.

### 2.4 Monosaccharide analysis by reversed phase UHPLC-ESI-MS/MS

#### 2.4.1 Hydrolysis and sample preparation

For each sample *~*100 µg of freeze-dried and dialyzed cell wall material were treated with either 200 µl 6 M HCl for 180 min at 100 °C (amino sugar analysis) or submitted to autoclaving (60 min, 121 °C) after the addition of 1 mL 2 M TFA (neutral sugar analysis). For solvent removal, samples were initially air-dried at 40 °C. Afterwards samples for neutral sugar analysis were washed once with 1 mL MeOH and twice with 2 mL MeOH (air-drying between and after washing steps). Samples for amino sugar analysis were washed only once with 2 mL MeOH and air-dried. Before derivatization, samples for both amino sugar and neutral sugar analysis were suspended in 400 µl H_2_O each. Subsamples of 25 µl were incubated with a mixture of 100 µl 25% (v/v) ammonia and 100 µl 1-phenyl-3-methyl-5-pyrazolone (PMP) for 100 min at 70 °C and 300 rpm before being cooled down to room temperature. Subsequently, samples were neutralized by adding 75 µl 100% acetic acid. Finally, excess PMP was removed by 3 steps of chloroform extraction (400 µl each). Before being measured by UHPLC-ESI-MS/MS, the aqueous phase (after extraction) was filtered through 3 kDa filters. Standards for all analyzed monomers (mannose, galactose, glucose, arabinose and glucosamine) were treated equally and included in every hydrolysis batch in the following amounts: 3 µg, 6.25 µg, 12.5 µg, 25 µg, 50 µg, 75 µg, 100 µg.

#### 2.4.2 Detection using UHPLC-ESI-MS/MS

**Supplementary Fig. S1:**
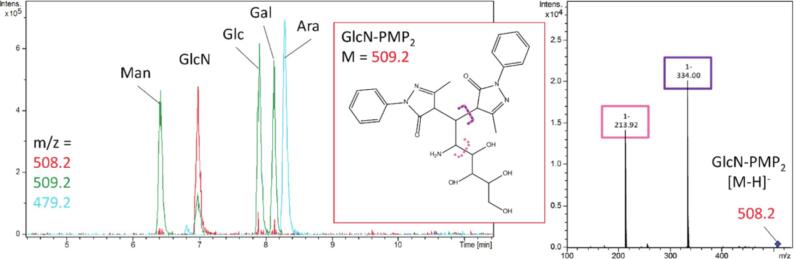
Reversed phase UHPLC-ESI-MS/MS analysis of PMP-derivatized monosaccharide-standards. (A) Extracted-ion chromatograms of the derivatized neutral sugars mannose-PMP2 (Man), glucose-PMP2 (Glc), galactose-PMP2 (Gal) and arabinose-PMP2 (Ara) as well as the amino sugar glucosamine-PMP2 (GlcN) (MS1, negative ion mode), (B) Example MS2 spectrum of glucosamine-PMP2. Fragmentation behavior of derivatized monosaccharide precursor ions is illustrated with GlcN-PMP2 as an example (A). The fragment with m/z = 213.9 is used for quantification.

Samples were measured on a Dionex Ultimate 3000RS UHPLC system (Thermo Scientific, Milford, USA) equipped with a Zorbax Eclipse Plus C18 column (1.8 µm, 2.1 mm × 100 mm; Agilent, Santa Clara, USA) and an Eclipse Plus guard column (1.8 µm, 2.1 mm × 5 mm; Agilent, Santa Clara, USA). The LC-system was coupled to an amaZon speed ESI-MSn detector (Bruker Daltonik, Bremen, Germany). PMP derivatized monosaccharides were separated based on a method previously described (Xu et al., 2018), using the following 14 min gradient elution profile: 0–1 min isocratic 95% A (95% H_2_O, 5% acetonitrile, 25 mM ammonium acetate, pH 7.8); 1–9 min linear from 5% to 25% B (5% H_2_O, 95% acetonitrile); 9–9.5 min linear from 25% B to 99% B; 9.5–12 min isocratic 99% B; column re-equilibration: 12–12.5 min linear from 99% B to 95% A; 12.5–14 min isocratic 100% A. 2 µl of sample were injected into the system and samples were measured in UltraScan mode and negative ion mode (Auto MS^2^) with the following parameters: capillary voltage 4000 V; plate offset voltage = 500 V; isolation width = 4 m z-1; nebulizer pressure = 1.03 bar. Dry gas = 8 L min^−1^; dry temperature = 200 °C ICC target = 100000; max. accumulation time 50 ms; CID fragmentation; For each derivatized monosaccharide (monosaccharide-PMP_2_), one of the most abundant fragment ions of the precursor ion [M−H]^−^ was used for quantification as previously described (Xu et al., 2018) (see Table 2). To minimize fragmentation of non-target molecules, we included only precursor masses in the following *m*/*z* ranges (Fig. S1): 474–484 (covering Arabinose-PMP_2_) and 503–514 (covering Mannose-PMP_2_, Glucosamine-PMP_2,_ Glucose-PMP_2,_ Galactose-PMP_2_).

**Table 2:** *m*/*z* values of precursor and fragment ions of derivatized monosaccharides.

Molecule	Precursor ion *m*/*z*	Fragment ion *m*/*z*	Retention time
Mannose-PMP_2_	509.19	214.975	~6.3 min–6.6 min
Glucosamine-PMP_2_	508.25	213.97	~6.9 min–7.2 min
Glucose-PMP_2_	509.19	214.975	~7.8 min–8.05 min
Galactose-PMP_2_	509.19	214.975	~8 min–8.25 min
Arabinose-PMP_2_	479.17	214.975	~8.2 min–8.5 min

#### 2.4.3 Data analysis

**Supplementary Fig. S2:**
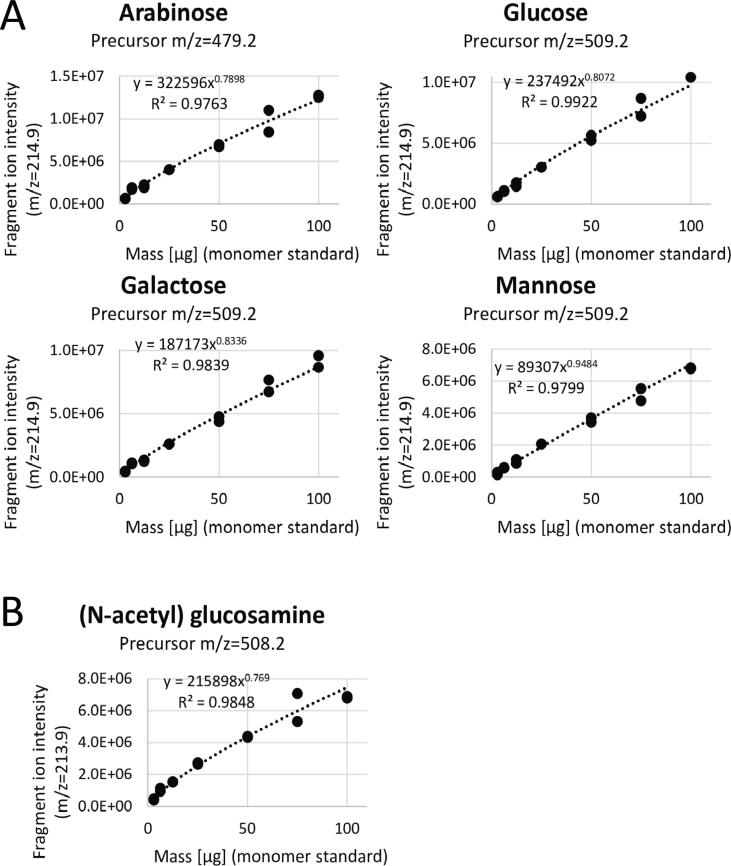
Example standard curves for the different monosaccharides. (A) neutral sugars (B) amino-sugars; N-acetylglucosamine is de-N-acetylated by HCl treatment.

Fragment ion intensities of given precursor ions were integrated over a pre-defined retention time window, allowing a mass error of 2 Da for both precursor and fragment ion *m*/*z*. See Table 2 for *m*/*z* values and retention times. Measurements of derivatized monosaccharide standards in different concentration were used to determine power calibration curves (Fig. S2). The µg amount of each monosaccharide per mg (dry weight) cell wall ($c_{\mathrm{mono}/CW}$) was calculated as follows: $c_{\mathrm{mono}/CW}=w_{\mathrm{CWF}}\frac{m_{\mathrm{mono}}w_{\mathrm{mono}}}{m_{\mathrm{CWF}}}$, where the mass fraction $w_{\mathrm{CWF}}$ is the mass of dialyzed cell wall fraction [mg] divided by the mass of total cell wall [mg] (dry weight). $m_{\mathrm{mono}}$ is the mass of a monosaccharide [µg] determined by mass spectrometry $m_{\mathrm{mono}}={\left(\frac{I}{a}\right)}^{\frac{1}{b}}$, with I = arbitrary intensity of the fragment ion; a and b are coefficients determined by fitting a power function to a measured series of monosaccharide standards. The weight fraction $w_{\mathrm{mono}}=\frac{M_{\mathrm{mono}}-M_{H2O}}{M_{\mathrm{mono}}}$, with $M_{\mathrm{mono}}$ = molar mass of monosaccharide, and $M_{H2O}$ = molar mass of water, is used to compensate for the gain of mass due to hydrolysis of glycosidic linkages in cell wall polysaccharides. The mass $m_{\mathrm{CWF}}$ is the dry weight [mg] of dialyzed cell wall fraction that was subjected to hydrolysis and derivatization. For further details, see supplementary Figs. S1 and S2.

### 2.5 Bioreactor cultivation

Controlled batch fermentations for *A. niger* strains MA234.1 and TLF39 were performed in 6.6L BioFlo bioreactors (New Brunswick Scientific), as previously described (Jørgensen et al., 2010). A batch of 21 L MM containing 0.75% D-glucose was made by adding 1 L filter-sterilized (0.2 µm pore) glucose (15.75% w/v) solution to a freshly autoclaved volume of 20 L MM (no carbon source) as described above. Allowing 1 day of dissolving and a check for contamination, 5 L MM 0.75% glucose was added to each bioreactor directly after autoclaving. Temperature, acidity and stir speed were set to and kept at 30 °C, pH 3 and 250 rpm, respectively. The pH was controlled by addition of titrants (2 M NaOH and 1 M HCl). Sparger aeration of 1 L/min was left on to allow oxygen saturation of the medium prior to inoculation. Next, aeration was set to headspace only and 1.5 mL 10% w/v Yeast Extract was added to the medium to promote homogeneous germination for the to-be-added spores. Subsequently, a total of 5x10^9^ (10^6^ sp/mL) spores were added to the medium using a concentrated spore solution. Germination time of approximately 4–5 h was maintained, preceding the addition of polypropyleneglycol P2000 anti-foam agent, increasing agitation to 750 rpm and changing aeration from headspace to sparger only (1 L/min). Oxygen, base and acid consumption were monitored and samples were taken at regular intervals to obtain biomass, culture filtrate and microscopy samples. Biomass was harvested by applying a vacuum over Whatman™ Glass Microfiber Filter (GF/C™) (diameter 47 mm, CAT No.1822-047, Buckinghamshire, UK). Samples were all quickly frozen in liquid nitrogen prior to storage at −80 °C. Biomass accumulation through time was gravimetrically determined by lyophilizing designated samples from the corresponding broth culture mass.

### 2.6 RNA isolation and RNA-sequencing

RNA was isolated from mycelial biomass samples obtained from batch cultivated *A. niger* strains MA234.1 and TLF39, using TRIzol (Invitrogen). RNA was purified afterwards with NucleoSpin RNA Clean-up kit (Macherey-Nagel) with DNase treatment. Concentration and quality of the RNA was determined using a NanoDrop 2000 spectrophotometer (Thermo Scientific) and by gel-electrophoresis, respectively. RNA samples were sent to Genome Québec for sequencing using the HiSeq4000 technology. Sequencing data is available under GEO accession number GSE142461.

### 2.7 Transcriptomic analysis

**Supplementary Fig. S3:**
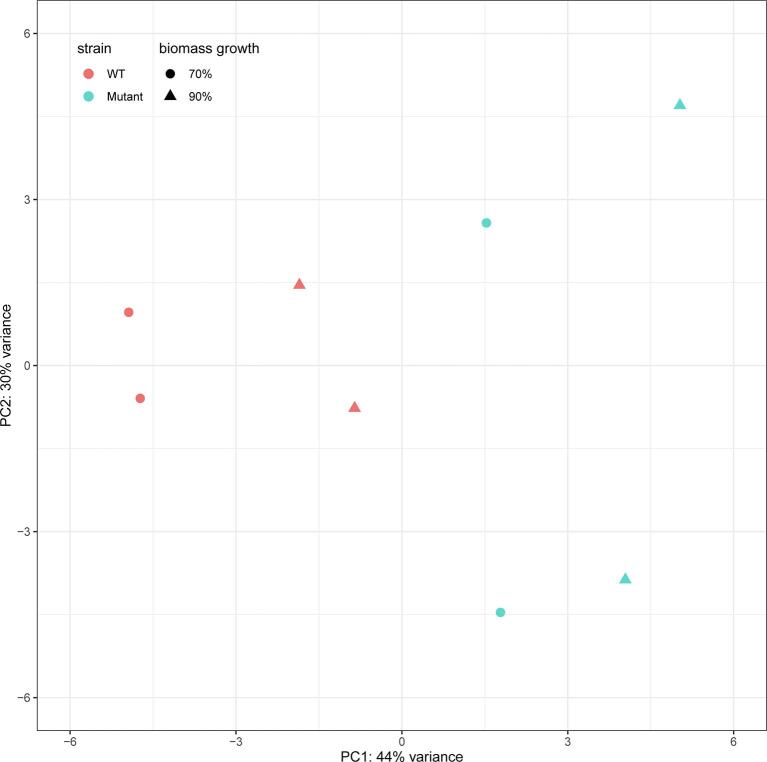
Principal component analysis of the gene expression data obtained by RNA sequencing. The RNA sequencing libraries were separated in the first-dimension axis according to the mutation of the *crh* genes.

**Supplementary Fig. S4:**
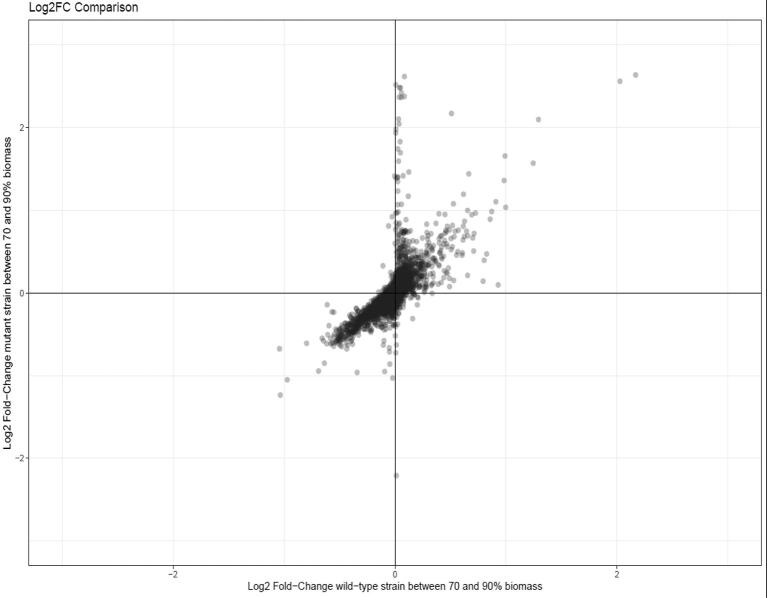
Correlation of fold-change between 70 and 90% biomass. Growth in the WT (x-axis) and the *crh* genes mutated strain (y-axis). Each dot represents a gene.

Raw RNA-seq read sets were retrieved from the Génome Québec’s Nanuq portal, and pre-processed with BBDuk from the BBTools package (https://sourceforge.net/projects/bbmap) to trim sequencing adapters and remove reads derived from PhiX and ribosomal RNA. The transcriptome of *A. niger* NRRL3 (v. 20140311) was retrieved from the *jgi* Genome portal (Aguilar-Pontes et al., 2018), and the libraries were mapped to the transcriptome using Salmon v0.14.1 (Patro et al., 2017). The libraries were imported in RStudio 1.2.5001 (RStudio: Integrated Development for R. RStudio, Inc., Boston, 2016) running R 3.6.1 (R Development Core Team 3.6.1., 2019) using txtimport v.1.12.3 (Soneson et al., 2016). Initial data exploration showed that the major source of difference between the libraries was due to the strain (Fig. S3), although the time-point of sampling was also an important factor to consider. However, both strains behaved in the same way between the 70% and 90% biomass time-points (Fig. S4). That is, there was no significant interaction between the 70 and 90% biomass growth and the mutation. Based on this information, we considered the biomass time-points as biological duplicates, thereby increasing the statistical power. Differential gene expression was assessed via pairwise comparisons using DESeq2 v1.24.0 (Love et al., 2014), using the design ~ biomass + mutation (*p*adj ≤ 0.05). Data was manipulated using tidyverse (Wickham, 2017a) and plotted in ggplot2 (Wickham, 2017b). Gene ontology enrichment (*p*adj ≤ 0.05) was performed with goseq v1.36.0 (Young et al., 2010). Updated gene length and Gene Ontology data for the NRRL3 genome was retrieved from the *jgi* Genome portal. The full code is available at github.com/gabrifc/TLF39_transcriptome.

### 2.8 Single and double gene knockouts

A deletion of *ugmA* was introduced in the seven-fold *crh* knockout strain (TLF39) using a split marker approach as described below. To target α-1,3-glucan synthesis, we disrupted both α-glucan synthase A (NRRL3_07454) and α-glucan synthase E (*agsE*, NRRL3_00248) separately, and constructed a double *agsA, agsE* knockout using CRISPR/Cas9 and repair fragments (see below).

#### 2.8.1 Split marker fragments

MA234.1 and TLF39 (Table 1) were transformed after protoplastation as described previously (Arentshorst et al., 2012). Using the split marker approach for single gene knockouts, entire ORFs were deleted by replacement with the hygromycin B selection marker (Arentshorst et al., 2015). Flanks were generated via PCR using N402 genomic DNA as template and primers as described in Primer Table. *AOpyrG* fragments were obtained from plasmid pAN7-1 (Punt et al., 1987) with primers as described in Primer Table. Fusion PCR was used to generate split marker fragments containing *AOpyrG.* Approximately 2 µg of DNA per flank was added to protoplasts for transformation. Transformation plates were incubated on MMS for 6 days at 30 °C. Transformed colonies were single streaked on MM twice for purification and were genotyped using diagnostic PCR (data not shown).

#### 2.8.2 CRISPR/Cas9 and repair DNA fragments

CRISPR/Cas9 plasmid transformations were performed after protoplastation as described previously (van Leeuwe et al., 2019). Plasmids were constructed using a pFC332 (*hph*) plasmid (Nødvig et al., 2015) backbone and repair fragment were generated using fusion PCR with N402 genomic DNA as template as described in Primer Table. For transformations 2 µg of Cas9-sgRNA plasmid with 2 µg of repair DNA fragment were used. Transformation plates were incubated on MMS with 200 µg/mL hygromycin for 7 days at 30 °C. Transformed colonies were single streaked on selectable MM with 100 µg/mL hygromycin to select for the presence of the Cas9-sgRNA plasmid. Next, a single colony was picked and transferred to non-selective MM medium to allow loss of the Cas9-sgRNA plasmid. A third streak of a single colony on both MM and MM with 100 µg/mL hygromycin uridine was performed as a control for loss of plasmid. DNA from plasmid-cured strains was isolated as described by Arentshorst et al. (2012), using mortar and pestle to grind the mycelium in liquid nitrogen. Genotypes were confirmed using diagnostic PCR to check for open reading frame removal.

## 3 Results

### 3.1 *Aspergillus niger* possesses seven *crh* gene orthologues

Crh enzymes have most extensively been studied in yeast, but are well conserved in fungal genomes (Arroyo et al., 2016). In *A. niger,* seven *crh* genes (*crhA* to *crhG*) have been annotated based on homology with both *CRH1, CRH2* and *CRR1* (Pel et al. (2007) and Table 3). Using SignalP 4.1 (Nielsen, 2017), N-terminal signal sequences were predicted for all seven enzymes. Aside from CrhF and CrhG, the Crh enzymes in *A. niger* were also predicted to contain Glycosylphosphatidylinositol (GPI) anchors by the PredGPI server (Pierleoni et al., 2008), whereas CrhF and CrhG were predicted to contain a single transmembrane helix (TMH) using TMHMM 2.0 (Krogh et al., 2001, Sonnhammer et al., 1998). Using ScanProsite (de Castro et al., 2006), we identified that all seven Crh enzymes carry a catalytic active site E-I-D-[WFL]-E, similar to the previously reported common catalytic motif (E-[ILV]-D-[IVAF]-[VILMF](0,1)-E), in which the first glutamic acid, central aspartic acid and final glutamic acid act as nucleophile, auxiliary residue and the general acid/base, respectively (Gaiser et al., 2006, Hahn et al., 1995, Johansson et al., 2004, Keitel et al., 1993). Additionally, all Crh enzymes carry the essential substrate-binding pocket GTIXWXGG motif for transglycosylation activity as reported in *Aspergillus fumigatus AfCrh1-5* (Fang et al., 2019), whereas CrhG was found to have another polar amino acid at the conserved second position of this motif (GNIXWXGG). In addition to these seven members of the GH16 family, six other GH16 enzymes are reported for *A. niger* in the CAZy database (NRRL3_06188, NRRL3_02000, NRRL3_02614, NRRL3_05937, NRRL3_01051 and NRRL3_05408). However, these do not show up as reciprocal BLAST hits with *CRH1, CRH2* and *CRR1*. Despite a GH16 active site in these GH16 enzymes (E-[IS]-D-[IV]-[ILV]-E), these non-Crh GH16 enzymes of *A. niger* do not have the consensus GTIXWXGG motif of Crh enzymes, as is also not the case for the yeast GH16 enzymes Kre6p and Skn1p.

**Table 3:** GH16 enzymes in *A. niger* and properties thereof.

NRRL3 ID	An ID	Gene	Protein length (AA)	GH16 catalytic motif*	Location catalytic motif	Consensus *crh* motif**	Location sugar-binding motif	Signal peptide	GPI anchor	TMH
NRRL3_10021	An11g01540	*crhA*	366	EIDWE	118–122	GTIDWAGG	215–222	yes	yes	0
NRRL3_04809	An07g07530	*crhB*	424	EIDFE	163–167	GTIEWAGG	266–273	yes	yes	0
NRRL3_04315	An07g01160	*crhC*	401	EIDLE	159–163	GTIEWAGG	262–269	yes	yes	0
NRRL3_02532	An01g11010	*crhD*	396	EIDWE	118–122	GTIEWAGG	216–223	yes	yes	0
NRRL3_01365	An13g02510	*crhE*	328	EIDWE	122–126	GTIEWAGG	220–227	yes	yes	0
NRRL3_07085	An16g02850	*crhF*	363	EIDWE	118–122	GTIVWAGG	216–223	yes	no	1
NRRL3_03998	An15g05350	*crhG*	443	EIDWE	117–121	GNIEWGGG	217–224	yes	no	1
NRRL3_06188	An02g00850	–	739	EIDIIE	140–145	n.d.	–	yes	yes	0
NRRL3_02000	An01g04560	*–*	432	EIDIIE	136–141	n.d.	–	yes	no	0
NRRL3_02614	An01g11970	*–*	484	EIDVLE	220–225	n.d.	–	no	no	1
NRRL3_05937	An02g03980	*–*	653	EIDVIE	438–443	n.d	–	no	no	1
NRRL3_05408	An02g10490	*–*	396	EIDVVE	212–217	n.d	–	no	no	0
NRRL3_01051	An14g05490	–	476	ESDIE	228–232	n.d.	–	yes	no	1

*Hits for E-[IS]-D-x-[VILMF](0,1)-E.

**Hits for G-[TN]-I-x-W-x-G-G.

n.d. (not detected).

### 3.2 Multi-condition expression data shows *crh* genes activity during different stages of development

In yeast, *CRH1* and *CRH2* are both expressed during vegetative growth, whereas *CRR1* is only induced upon sporulation. This suggests diversification of Crh enzymes to fulfill a role in different stages of growth. As a first step in our studies on the Crh gene family in *A. niger* we analyzed expression levels of *crh* genes in a gene-expression dataset, covering 155 different cultivation and developmental conditions (Paege et al., 2016, Schäpe et al., 2019). We previously reported on a sub-set of these conditions where we looked into *crh* expression under plate growth conditions and during exponential growth from liquid fermentation (van Leeuwe et al., 2019). Here, we expanded this study for different growth and developmental phases, including germination (Novodvorska et al., 2013, van Leeuwen et al., 2013), exponential vegetative growth, starvation and sporulation conditions (Jørgensen et al., 2010, van Munster et al., 2015) as well as sclerotium formation (Jørgensen et al., 2020), spore maturation (Teertstra et al., 2017) and exposure to cell wall disturbing compounds (Meyer et al., 2007, Fiedler et al., 2014). An overview of these gene expression data expressed as a percentage of actin expression is shown in Table 4.

**Table 4:** Microarray expression data of *crh* genes (as percentage of actin expression).

Condition	*crhA*	*crhB*	*crhC*	*crhD*	*crhE*	*crhF*	*crhG*	Reference
Germination of conidia								
0 h	0.5	2	1	1	0.9	3.3	0.5	Novodvorska et al., 2013
1 h	0.5	6	1.5	6	1	0.8	0.5	
2 h	0.5	21	10	9	1.5	1.1	0.5	
4 h	0.5	18	21	2	1.5	0.8	0.5	
6 h	0.5	12	13	2	1.5	1.2	0.5	

Germlings (5 h) response to either Caspofungin (CA), Fenpropimorph (FP), Aureobasidin A (AbaA) or FK506, 1 h post-incubation								
CA/FP ctrl	0.5	7.5	21	5.6	1.1	0.9	0.4	Meyer et al., 2007, Fiedler et al., 2014
CA	0.6	14	38	4.4	1.2	1.9	0.3	
FP	0.6	7.1	22	8.9	1.4	1	0.3	
AbaA ctrl	0.7	15	36	4.5	1.9	1.3	0.5	
AbaA	0.9	20	46	3.7	1.9	1.6	0.4	
FK506 ctrl	1.3	21	34	3.6	1.8	2.1	0.6	
FK506	0.8	14	34	3.3	1.5	2.5	0.5	

Retentostat culture of N402 approaching zero growth rate on maltose								
0 days	1	4.3	2.8	20	1.6	1	0.2	Jørgensen et al., 2010
2 days	1.5	4.5	1.6	39	1.1	2.1	0.1	
8 days	0.6	3.2	1.6	20	1.3	2	0.2	

Carbon starvation and sporulation onset on maltose								
N402 exponential growth	0.9	4.4	2.4	18	1.4	1.2	0.4	van Munster et al., 2015
*ΔbrlA* exponential growth	2.2	6	5	9	3	1.5	0.5	
*ΔflbA* exponential growth	2.8	21	18	11	1.5	3.5	0.5	
							
N402 carbon starvation day 1	1.9	3.3	0.9	87.8	0.9	0.8	0.4	
*ΔbrlA* carbon starvation day 1	1	3	2	17	1.5	1.1	0.5	
*ΔflbA* carbon starvation day 1	1	10	10	25	1.2	1.1	0.5	
N402 carbon starvation day 3	1	3.6	2	57.3	0.9	3.7	0.4	
*ΔbrlA* carbon starvation day 3	1.5	8	6.8	7	1.5	1.5	0.5	
*ΔflbA* carbon starvation day 3	0.7	9	6	13.5	1.5	1.5	0.5	
N402 carbon starvation day 6	0.5	4.3	2.4	38.9	0.8	2.5	0.3	
*ΔbrlA* carbon starvation day 6	0.9	7	10	2	1.5	1	0.5	
*ΔflbA* carbon starvation day 6	1	8.5	11	7.5	1.7	1.5	0.5	

Sclerotia formation on glucose plates								
*ΔsclB* mycelium	2	8.2	8.5	5	1.2	3.2	0.5	Jørgensen et al., 2020
*ΔsclB* sclerotia	10	3.4	1.5	0.9	0.9	1.5	0.5	

From the analysis it is clear that during conidial germination, in particular *crhB* and *crhC* are expressed upon swelling, initial germ tube formation, and early branching (t = 1–6 h). In non-germinated, resting conidia (t = 0 h), we find very low levels of *crh* gene transcripts, the highest being *crhF* (Novodvorska et al., 2013). These low levels of *crh* gene transcript are in congruence RNA-seq data from maturing spores (Teertstra et al., 2017), and also show the highest relative number of transcripts for *crhF* followed by *crhB* (Table S1), similar as found in Table 4 (t = 0 h). Upon exposure to antifungal compounds caspofungin (β-1,3-glucan synthase inhibitor, CA) and aureobasidin A (sphingolipid biosynthesis inhibition, AbaA) in germlings, both *crhB* and *crhC* were reported to be higher than in control (no drug added) experiments (Fiedler et al., 2014, Meyer et al., 2007), whereas only *crhD* was induced in the presence of fenpropimorph (sterol biosynthesis inhibition, FP) (Table 4).

During exponential growth, *crhD* is the highest expressed *crh* gene. Nonetheless, both *crhB* and *crhC* are also expressed. Carbon starvation showed to induce the expression of *crhD* even further. Because the increase of *crhD* is not observed during carbon starvation of non-sporulating *ΔbrlA* and *ΔflbA* mutants, the induction of *crhD* is likely to relate to spore formation. No other *crh* genes were specifically expressed during the starvation time points, suggesting that *crhD* is the main *crh* gene involved in spore formation and maturation. We noticed the high expression of *crhB* and *crhC* in the *ΔflbA* mutant which might explain the different cell wall morphology observed in the *ΔflbA* mutant (Krijgsheld et al., 2013). The involvement of *crh* genes in spore development was also analyzed by examining gene expression in retentostat cultures. During the later time points, massive conidiation was observed evidenced by black pigmentation in the effluent (Jørgensen et al., 2010), but no apparent strong induction of any of the *crh* genes was found, again with the exception of *crhD*.

*A. niger* mutants have been isolated to form sclerotia (Jørgensen et al., 2011). During sclerotium formation, *crhA* is specifically induced, suggesting a role for *crhA* during sclerotium development. Finally, in the study by Park et al, it was observed that *crhE* was the only significantly induced (7.51 FC) *crh* gene upon cell wall stress in the galactofuranose cell wall deficient *ΔugmA* (UDP-galactopyranose mutase A) mutant (Park et al., 2016).

In conclusion, the expression analysis suggests that different *crh* genes are expressed under different conditions. Most noticeable is the expression of *crhA* during sclerotia formation, *crhB* and *crhC* during germination, *crhD* during vegetative growth and sporulation and *crhE* during cell wall stress caused by the absence of cell wall galactofuranose. Expression of *crhF* and especially *crhG* were low across all tested conditions, though *crhF* may be involved in spore maturation.

### 3.3 Cell wall integrity is not disturbed by loss of the *crh* gene family

Loss of *CRH* genes in *S. cerevisiae* and *C. albicans* has previously been reported to affect cell wall integrity when exposed to the cell wall stressing compound CR, but displayed normal growth under non-stressing conditions (Rodriguez-Pena et al., 2000, Pardini et al., 2006). A quintuple *crh* knockout strain in *A. fumigtus* was recently shown to have a much lesser effect on growth when exposed to CR compared to yeasts (Fang et al., 2019). In line with the results observed for *A. fumigatus*, we also showed that single knockouts strains of either *crhA, crhB, crhC, crhD, crhE, crhF* or *crhG*, as well as a double, triple, quintuple and seven-fold knockouts strains of *crhA-G* (TLF39), were unaffected by the presence of cell wall disturbing compounds CR and CFW (van Leeuwe et al., 2019). Additional assays were conducted to assess the impact of a full *crh* gene family knockout in this study, and include sensitivity assays towards SDS (cell wall/cell membrane disturbing), H_2_O_2_ (oxidative stress), tunicamycin (N-glycosylation, unfolded protein response) and CA. However, both the wild type and the seven-fold deletion strain (*ΔcrhA-G*) showed the same levels of sensitivity towards all these tested compounds (data not shown).

**Fig. 1:**
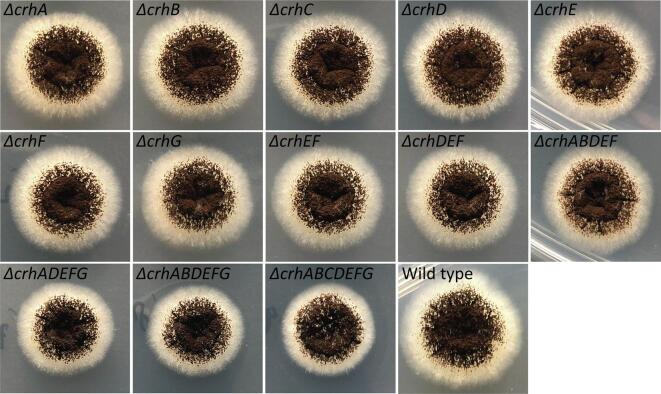
Colony morphology of *crh* mutants and wild type (MA234.1) on MM. All strains were grown equally as described in Section 2.1, and were spotted to contain 10^4^ spores. Plates were incubated at 30 °C for 3 days.

In addition to these cell wall stress assays, closer examination of colony morphology during vegetative growth was carried out showing that there is a minor difference between wild type (MA234.1) and TLF67 (*ΔcrhADEFG*), TLF68 (*ΔcrhABDEFG*) and TLF39 (*ΔcrhABCDEFG*) in radial growth, causing a slightly more compact colony, but not for single, double, triple and quintuple (*ΔcrhABDEF*) mutants (Fig. 1). This result indicated that Crh activity is at least to some extent involved in surface growth behavior, but no differences in hyphal thickness, branching or other aberrant growth behavior were observed when looking into the edges of these colonies.

### 3.4 Genome-wide expression profiling reveals high similarity between mutant and wild type

**Supplementary Fig. S5:**
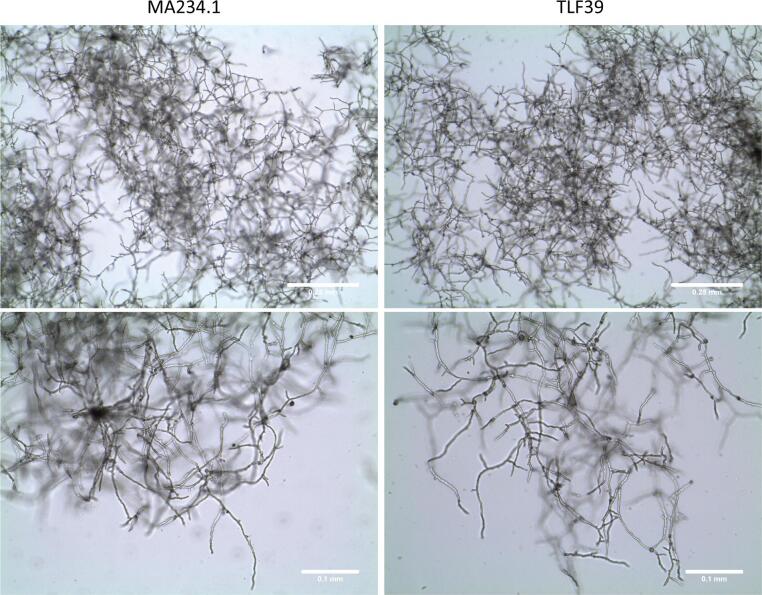
Hyphal and pellet morphology of glucose batch cultures during exponential growth. Wild type, MA234.1 (left) and *ΔcrhA-G*, TLF39 (right). 70% biomass.

To further study the effect of deleting the *crh* family, we performed controlled batch fermentations followed by genome wide gene expression analysis by RNA-sequencing in the seven-fold *crh* mutant (TLF39) and the wild type strain (MA234.1). Both strains were cultivated in 0.75% glucose batch-fermentations in bioreactors as described in Section 2.5. Dry weights of fermentations were used to determine the maximal specific growth rate. Both MA234.1 (0.224 ± 0.013 · h^−1^) and TLF39 (0.219 ± 0.005 · h^−1^) were found to exhibit equal growth rates, maximum biomass acquisition (MA234.1 g_DW_ 6.4 ± 0.5 · kg^−1^ and TLF39 g_DW_ 6.4 ± 0.15 · kg^−1^) and similar base consumption (data not shown). In addition, morphological analysis of culture samples showed that both wild type and mutant display a very similar growth phenotype (Fig. S5).

**Supplementary Fig. S6:**
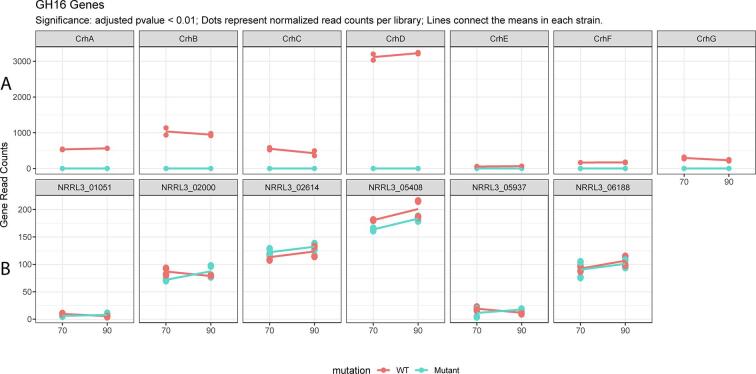
Normalized gene read counts for the *crh* and other GH16 genes. WT (red) and mutant (teal) libraries. While there is a complete decrease of detectable expression of the mutated genes, the expression of other GH16-family genes is not affected.

RNA was isolated from culture samples in the exponential growth phase at both 70% and 90% of the maximum attained biomass. Duplicate batch-culture cultivations for both wild type and mutant were used to obtain a total of 8 RNA-sequencing samples. RNA sequencing was performed as described in Section 2.6, followed by transcriptomic analysis (Section 2.7). We found 230 transcripts—approximately 2% of the 11,846 total transcripts—to be differentially expressed between the mutant strain and the wild type (adjusted *p*-value ≤ 0.05) (Table S2). First, we found that wild type expression of *crh* genes is in line with previous transcript levels observed in Table 4, where *crhD* is most highly expressed during exponential growth, followed by *crhB, crhC* and *crhA* (Fig. S6). Contrarily in the mutant, the deleted *crh*-family genes were no longer expressed, thus validating their knockout, whereas for the other GH16 family members no significant difference was observed between the mutant and the wild type (Fig. S6). In accordance to this result, most of the observed variation between mutant and wild type was explained by the difference in expression in these 7 *crh* genes.

Gene Ontology (GO) analysis of the differentially expressed genes suggests very few processes are significantly affected between wild type and mutant (Table S3). Three enriched processes pertaining to cell wall biosynthesis (licheninase activity (GO:0042972), hydrolase activity/hydrolyzing O-glycosyl compounds (GO:0004553), and carbohydrate metabolic process (GO:0005975)), were identified based solely on the differential expression of the *crh* genes present in these three GO categories. Therefore, we also decided to specifically analyze differential gene expression in the datasets of genes involved in cell wall biosynthesis. In this manual search we used a list of all annotated cell wall biosynthetic genes described by Pel et al. (2007) and an overview of all cell wall integrity (CWI) response-related genes as previously described (Meyer et al., 2007, Park et al., 2016). Significantly upregulated and significantly downregulated cell wall biosynthetic genes are shown in Table 5, Table 6, respectively. For the upregulated genes, the highest fold change was found for an aldose 1-/glucose-6-phosphate 1-epimerase family protein (NRRL3_10372), responsible for interconversion between α-glucose and β-glucose. Noticeably, this gene is part of a previously uncharacterized putative secondary metabolite gene cluster for which the expression of all genes was upregulated in TLF39 (NRRL3_10368 – NRRL3_10375), suggesting that this gene is not related to a genuine cell wall related function. As mentioned previously, besides the *crh* gene family, no differential expression of other GH16 enzymes was found and no clear signs for cell wall stress were observed. We only found slight up-regulation for both *gfaB* and *pirA,* yet no induction of other CWI pathway related genes such as *agsA*, *phiA*, *gfaA* (CWI genes highlighted in Table S2), confirming that the CWI pathway is not activated in the seven-fold deletion strain.

**Table 5:** Putative cell wall biosynthetic related genes upregulated in the TLF39 mutant. Listed reads are the average of 70% and 90% biomass DESeq2 normalized (by library and gene length) reads.

Gene ID	Gene name	Description	WT reads	Mutant reads	FC	P-adj
NRRL3_10372		aldose 1-/glucose-6-phosphate 1-epimerase family protein	607	2045	3.27	5.38E-09
NRRL3_00240		carbohydrate-binding module family 50 protein (LysM)	30	78	2.64	4.35E-04
NRRL3_02950	*pirA*	putative cell wall protein with internal repeats	4982	7950	1.48	3.16E-04
NRRL3_00771	*cwpA*	glycosylphosphatidylinositol-anchored cell wall mannoprotein	322	464	1.44	5.47E-05
NRRL3_04222		carbohydrate-binding module family 50 protein (LysM)	156	221	1.41	2.60E-03
NRRL3_00251		cell-wall synthesis Kre9/Knh1 family protein	1115	1541	1.38	2.62E-14
NRRL3_04221		chitinase-like protein	138	189	1.37	1.99E-02
NRRL3_00249	*agtA*	GPI-anchored α-glucanosyltransferase	1811	2311	1.28	1.31E-07
NRRL3_08822		cell-wall synthesis Kre9/Knh1 family protein	1316	1675	1.27	2.00E-08
NRRL3_08347	*gfaB*	glutamine-fructose-6-phosphate aminotransferase	1001	1224	1.23	1.41E-02

**Table 6:** Putative cell wall biosynthetic genes downregulated in the TLF39 mutant. Listed reads are the average of 70% and 90% biomass DESeq2 normalized (by library and gene length) reads.

Gene ID	Gene name	Description	WT reads	Mutant reads	FC	P-adj
NRRL3_02532	*crhD*	glycoside hydrolase family 16 protein	3170	0	∞	4.71E-35
NRRL3_04809	*crhB*	glycoside hydrolase family 16 protein	991	0	∞	4.32E-30
NRRL3_10021	*crhA*	glycoside hydrolase family 16 protein	548	0	∞	4.27E-26
NRRL3_04315	*crhC*	glycoside hydrolase family 16 protein	489	0	∞	3.36E-25
NRRL3_03998	*crhG*	glycoside hydrolase family 16 protein	262	0	∞	4.13E-21
NRRL3_07085	*crhF*	glycoside hydrolase family 16 protein	168	0	∞	1.90E-18
NRRL3_01365	*crhE*	glycoside hydrolase family 16 protein	60	0	∞	9.59E-13
NRRL3_08490		carbohydrate-binding module family 63 protein (expansin-like)	1578	1178	−1.34	2.43E-07
NRRL3_06700	*dfgG*	mannan *endo*-1,6-alpha-mannosidase	781	644	−1.21	1.31E-02
NRRL3_00523	*ctcA*	chitinase-like protein	18,371	15,208	−1.21	2.44E-03
NRRL3_03954		alpha-1,3-mannosyltransferase-like protein	1079	921	−1.17	1.22E-02
NRRL3_07862	*cfcC*	chitinase	1570	1372	−1.15	1.34E-02
NRRL3_04653	*chsA*	chitin synthase	1527	1356	−1.13	2.85E-02

### 3.5 Cell wall fractionation shows identical composition between wild type and mutant

**Fig. 2:**
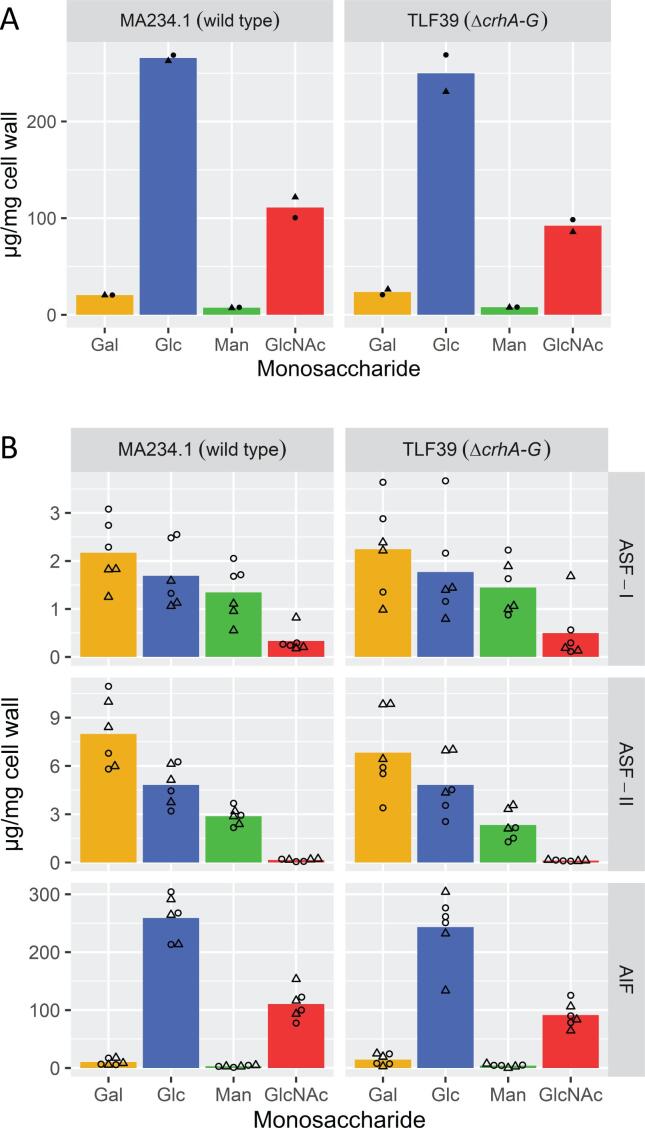
Monosaccharide cell wall composition of wild type strain and the seven-fold *crh* mutant (*ΔcrhA-G*). Cell wall isolation, fractionation, hydrolysis, monosaccharide detection and data processing are described in Sections 2.3 and 2.4. Measured monosaccharides include galactose (Gal), glucose (Glc), mannose (Man) and *N*-acetyl-glucosamine (GlcNAc), respectively shown as yellow, blue green and red colored bars. Fractions were obtained from two biological replicate bioreactor (BR) cultivations (maximum biomass) of both wild type (MA234.1), BR_TvL_15 and BR_TvL_18, and seven-fold *crh* mutant (TLF39), BR_TvL_16 and BR_TvL_17. Individual cell wall fractions are alkali soluble fraction I (ASF-I), alkali soluble fraction II (ASF-II) and alkali insoluble fraction (AIF). (A) Total monosaccharide composition per total cell wall dry weight (µg/mg cell wall) from combined fractions (Table S4). Data points are shown as either filled circles (BR_TvL_15 and BR_TvL_16) or filled triangles (BR_TvL_17 and BR_TvL_18), and represent averages of technical triplicate measurements per biological replicate. (B) Monosaccharide composition of each separate fraction as part of the total cell wall dry weight (µg/mg cell wall). Data points are indicated as either empty circles (BR_TvL_15 and BR_TvL_16) or empty triangles (BR_TvL_17 and BR_TvL_18), and represent technical triplicates per biological sample. (For interpretation of the references to color in this figure legend, the reader is referred to the web version of this article.)

Previous results showed no increased sensitivity towards to cell wall disturbing compounds and no indications for a cell wall stress response, exemplified by lack of induction of *agsA* and *gfaA* in the seven-fold *crh* mutant. Notwithstanding these results, we analyzed whether the seven-fold deletion affected the cell wall composition. To do so, we isolated cell walls from maximum biomass samples of both wild type (MA234.1) and seven-fold *ΔcrhA-G* mutant (TLF39), using aforementioned batch fermentation cultures. Cell walls were fractionated using a 5% (w/v) KOH and 24% (w/v) KOH alkali treatment (see Section 2.3.2) to obtain alkali soluble fraction I (ASF-I) and alkali soluble fraction II (ASF-II), respectively. Residual cell wall material after fractionation represents the alkali-insoluble fraction (AIF). ASF-I and ASF-II were dialyzed and weighed. Relative amounts of recovered cell wall dry weight per fraction were found to be of equal proportion for both MA234.1 and TLF39 (Table S4). The unaccounted fraction after recovery is likely attributable to the presence of both (cell wall) proteins and cell debris in the fractionated samples, and partially because of loss of material during transfer in fractionation and dialysis. Next, each fraction was hydrolyzed with either TFA or HCl (see Section 2.4.1), followed by derivatization and analysis to detect both neutral and amino sugars with UHPLC-ESI-MS (Section 2.4.2). The total cell wall composition as the sum of all fractions is displayed as relative amounts of sugar monomers per mg cell wall prior to fractionation, in Fig. 2A. Here, we found that there is no difference in the cell wall sugar composition between MA234.1 and TLF39 for galactose, glucose, mannose and (*N*-acetyl) glucosamine (arabinose was not detected). As β-1,3-glucan has been reported to become alkali insoluble due to its covalent linkage with chitin in the wild type cell wall (Hartland et al., 1994, Mol and Wessels, 1987, Sietsma and Wessels, 1979), we specifically looked in detail whether deletion of the *crh* genes resulted in a shift of the amount of β-1,3-glucan in the AIF to the ASFs of the cell wall, as shown before in yeast (Rodríguez-Peña et al., 2000). The AIF, shown in Fig. 2B, showed that the relative difference between MA234.1 and TLF39 of both glucose and chitin is slightly lower in TLF39. Additionally, we did not observe a significant increase in glucose in either ASF-I or ASF-II fractions (Fig. 2B), indicating the lack of *crh* genes neither affected the cell wall composition nor the extractability of glucans out of the AIF.

### 3.6 The *crh* gene family is relevant for cell wall integrity in the absence of galactofuranose and α-glucan biosynthesis

Phenotypic analysis, transcriptional data and cell wall composition analysis revealed that the *crh* gene family appears dispensable for growth and does not lead to reduced integrity of the cell wall itself. However, to assess the importance of Crh enzymes in strains with reduced cell wall integrity, we introduced a mutation causing galactofuranose deficiency (Δ*ugmA*) which was previously reported to have reduced integrity of the cell wall, accompanied by increased chitin levels (Damveld et al., 2008, Park et al., 2016). Cell wall chitin compensation has also been reported to occur by deletion of α-1,3-glucan synthases in *A. fumigatus* (Henry et al., 2012) and *A. nidulans* (Yoshimi et al., 2013). We hypothesized that disturbed cell wall integrity involving a chitin compensatory response also increases the importance of Crh facilitated chitin cross-linking. Additionally, we wanted to assess the role of Crh enzymes in relation to both chitin-α-1,3-glucan interaction and chitin to β-glucan cross-linking. Therefore, knockouts of *ugmA*, *agsE*, *agsA* and double *agsA/E* were performed in both wild type (MA234.1) and seven-fold *crh* mutant (TLF39) genetic backgrounds. All strains were verified by using diagnostic PCR (data not shown).

**Fig. 3:**
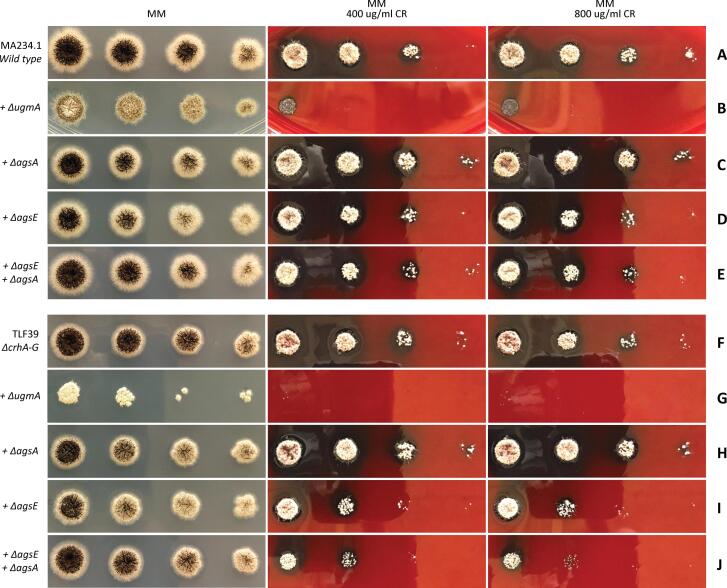
Growth morphology and Congo Red (CR) sensitivity assay. (A) Wild type (MA234.1) and introduced deletions (B) *ΔugmA* (MA613.1), (C) *ΔagsA* (TLF93), (D) *ΔagsE* (TLF94) and (E) *ΔagsA, ΔagsE* (TLF95) on the top five rows. (F) TLF39 (*ΔcrhA-G*) and the introduced deletions (G) *ΔugmA* (TLF70), (H) *ΔagsA* (TLF96), (I) *ΔagsE* (TLF97) and (J) *ΔagsA, ΔagsE* (TLF98) on the bottom five rows. Left column shows growth on MM, middle column shows growth on MM with 400 µg/mL CR and right column shows growth on MM with 800 µg/mL CR. All strains were spotted to contain equal amounts of spores from left to right: 10^4^, 10^3^, 10^2^ and 10^1^ spores. Plates were incubated at 30 °C for 65 h. (For interpretation of the references to color in this figure legend, the reader is referred to the web version of this article.)

**Supplementary Fig. S7:**
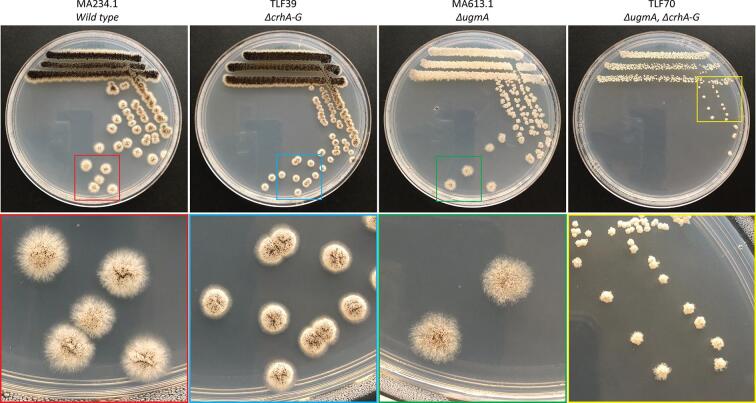
Growth of individual colonies on minimal medium (MM). Wild type (MA234.1), TLF39 (*ΔcrhA-G*), MA613.1 (*ΔugmA*) and TLF70 (*ΔugmA*, *ΔcrhA-G*) were single streaked on MM and were incubated at 30 °C for 3 days.

To assess the level of cell wall integrity, both wild type (MA234.1) and *ΔcrhA-G* (TLF39) strains with additionally introduced knockouts (either *ΔugmA*, *ΔagsE*, *ΔagsA* or *ΔagsA/E*) were exposed to CR, shown in Fig. 3. On MM, the *ΔugmA* (Fig. 3B) mutant grows and sporulates poorly compared to the wild type strain (Fig. 3A). Introduction of *ΔugmA* in the seven-fold *crh* mutant (TLF70, lane G) has an even more drastic effect on both colony size and sporulation compared to both *ΔugmA* (Fig. 3B) and *ΔcrhA-G* (Fig. 3F). It became evident that there is an additive effect of removing cell wall galactofuranose in the background of the seven-fold *crh* gene family knockout on both colony morphology and sporulation, by comparing individual colonies of *ΔugmA* with *ΔcrhA-G/ΔugmA* (Fig. S7). Additionally, Fig. 3 shows that sensitivity towards CR, while already much lower for *ΔugmA* compared to wild type, was also exacerbated in the background of the seven-fold *crh* mutant.

Contrarily to galactofuranose deficiency, neither growth nor sporulation defects were observed on MM by deleting either *agsA*, *agsE* or in the *agsA/agsE* double mutant in both wild type (Fig. 3C, D and E, respectively) and seven-fold *crh* mutant backgrounds (Fig. 3H, I and J, respectively). However, a knockout of *agsE* was found to increase CR sensitivity in the seven-fold *crh* mutant (Fig. 3F), but not in the wild type (Fig. 3A). Moreover, a double knockout of both *agsA* and *agsE* in the seven-fold *crh* mutant (Fig. 3J) resulted in an even more sensitive CR phenotype. The seven-fold *crh* mutants lacking either α-glucan or galactofuranose have also been tested for sensitivity towards CFW, CA and SDS. Similar to CR sensitivity, we found that double mutants were more sensitive to CFW. Susceptibility to both CA and SDS was identical between strains.

## 4 Discussion

The fungal cell wall matrix is a complex structure that shows large diversity among different species. It is subjected to continuous changes during growth and development, but also in response to environmental cues, mycelial age, available nutrients and cultivation conditions, hypoxia and other stresses (Free, 2013, Lord and Vyas, 2019, Pochanavanich and Suntornsuk, 2002). It is well established that the β-1,3-glucan-chitin core is the load-bearing structure of the cell wall and is essential for survival (Gow et al., 2017). As such, it is under continuous monitoring to ensure its integrity. Despite this importance, relatively few studies have investigated the covalent assembly of the β-1,3-glucan-chitin complex that was first shown to be facilitated by the Crh enzymes in *S. cerevisiae* (Rodríguez-Peña et al., 2000). Since then, Crh enzymes have been characterized to be solely responsible for this cross-linking activity and recently, advances have been made to investigate and characterize the role of these enzymes in filamentous fungi (Fang et al., 2019, van Leeuwe et al., 2019). Notwithstanding the same enzymatic activity of Crh enzymes in both yeasts and filamentous fungus *A. fumigatus* (Fang et al., 2019), the cell wall sensitivity phenotypes of *crh* gene family knockouts in *Aspergillus* species do not resemble results obtained in yeast species. In this study, we have investigated the impact of a seven-fold knockout of the *crh* gene family in *A. niger* in more detail.

First off, we investigated an existing, multi-condition expression data set for transcriptional activity of *crh* genes. Analysis of transcriptional activity revealed three majorly active members, namely *crhB, crhC* and *crhD*, whereas the remainder of the family members are expressed at much lower levels under all conditions tested. In addition to vegetative growth, we observed that *crhD* is likely to be involved during sporulation, yet we did not observe a reduction in sporulation activity in either single *crhD* or seven-fold *crh* gene family knockout strains (Fig. 1). Neither did we observe a difference in heat sensitivity of spores (Seekles and van Leeuwe, data not shown). Both *crhB* and *crhC* were found to be expressed upon germination, but neither germination nor growth rate were affected by the loss of the *crh* gene family. In addition, *crhB, crhC* and *crhD* were all shown to be induced upon exposure to differing cell wall stressing compounds, but appear to be dispensable for cell wall integrity based on previous sensitivity assays (van Leeuwe et al., 2019) and data presented here.

Regarding lowly expressed *crh* genes, *crhA* showed induced expression in sclerotial structures compared to the surrounding mycelium. This may suggest CrhA to play very specific role in this type of structural development, whereas expression levels of all other *crh* genes were relatively low (Table 4, Jørgensen et al., 2020). A single deletion of *crhA* (TLF57) and the seven-fold *crh* mutant (TLF39) were both tested for sclerotium formation and compared to sclerotium formation in the wild type. Both mutants and wild type were able to produce sclerotia, suggesting a non-essential role of *crh* genes during sclerotia formation (data not shown). Despite poor expression in all microarray data, we did find expression of *crhA*, *crhF* and *crhG* during vegetative growth in our batch fermentations using RNAseq. In addition to observed expression of *crhG* in *A. niger*, we also found that the introduction of a *crhG* deletion (combined with deleting either *crhA* or *crhA* and *crhB* simultaneously) in *ΔcrhDEF,* resulted in a small but significant compact colony phenotype (Fig. 1), but not as a single *ΔcrhG* knockout. Despite minor differences in growth on plate, we were not able to identify such changes in liquid conditions during submerged batch fermentations because of Crh loss, nor did we find expressive differences in pellet morphology. For now, the complexity of these genetic interactions as to when and whether other combinations of *crh* knockouts give rise to this compact phenotype have not been investigated and mechanisms of redundancy towards submerged growth remain unknown.

Due to the seemingly expendable nature of *crh* genes in *A. niger*, we performed RNA sequencing to assess whether the seven-fold *crh* deletion triggered a genome-wide transcriptional response. First, we did not find high fold changes in differentially expressed genes. Specifically, some cell wall related enzymes showed small fold-changes (maximum 3.27 < FC > -1.34), and no clear evidence of CWI pathway induction was observed upon deletion of the seven *crh* genes. Furthermore, absence of Crh enzymes in the seven-fold *crh* deletion mutant did not trigger in a transcriptional response of other non-Crh GH16 enzymes in *A. niger*. Recently, a non-Crh GH16 family member of *Trichoderma harzianum, gluc31* described as an *endo*-β-1,3-glucanase, was shown to have a role in cell wall biogenesis (Ribeiro et al., 2019). The authors argue that a deletion of *gluc31* causes thicker cell walls and a cell wall compensatory response by increased glucan and chitin deposition. A BLAST search with *gluc31* in the *A. niger* genome does not yield clear orthologous hits, the closest orthologue being a GH16 encoding gene (NRRL3_02000) with only 14% query coverage, 48.84% identity. A closer related species to *T. harzianum* and a Sodariomycete, *N. crassa*, revealed a BLAST-based orthologue NCU04431 to *gluc31* (96% query coverage, 56.74% identity), also encoding a GH16 member. A knockout of NCU04431 was recently shown to exhibit a wild type growth morphology (Patel and Free, 2019), which may suggest that *gluc31* only plays a specific role in cell wall biogenesis of *T. harzianum*. Taken together with transcriptional data from *A. niger*, we do not expect that other non-Crh GH16 enzymes of *A. niger* act in chitin to glucan transglycosylation.

Lack of differential gene expression relating to cell wall biogenesis suggests that the CWI pathway is not induced. However, as mentioned above, some cell wall related enzymes showed small fold-changes in the seven-fold *crh* mutant. These upregulated genes encode two Kre9/Knh1 like proteins, an α-glucanosyltransferase (*agtA*), cell wall proteins *cwpA* and *pirA,* and two LysM (CBM50) binding domain proteins. CBM50 binds with high affinity to chitin, but both proteins identified here (NRRL3_00240: 1 CBM50 domain, and NRRL3_04222: 5 CBM50 domains) lack catalytic domains. Lack of catalytic domains can be indicative of effector proteins that can either protect against chitinolytic activity or capture chitooligosaccharides that are released from the cell wall (Akcapinar et al., 2015). Previously, the cell wall protein *pirA* was identified to be induced upon caspofungin exposure, causing cell wall stress (Meyer et al., 2007), albeit at much higher levels (FC 23.03) than observed in the seven-fold *crh* mutant (FC 1.48). The *cwpA* gene was also found to be upregulated in the seven-fold *crh* mutant (FC 1.44), but in contrast to *pirA*, *cwpA* was not found to be induced upon cell wall stress. However, a deletion of *cwpA* was reported affect cell wall integrity, indicated by increased sensitivity towards CFW (Damveld et al., 2005a). CwpA belongs to the class of GPI anchored cell wall proteins (GPI-CWP). GPI-CWPs are cross-linked to cell wall β-1,3-glucan via a flexible β-1,6-glucan moiety through a phosphodiester linkage with the GPI-anchor (Frieman et al., 2002, Kapteyn et al., 1996, Kollár et al., 1997, Lu et al., 1995, Lu et al., 1994). The presence of β-1,6-glucosylated cell wall proteins has been reported in *A. niger* (Brul et al., 1997), yet presence of linear β-1,6-glucan chains such as in *S. cerevisiae* remains elusive in filamentous fungi, and is known to be absent in both *A. fumigatus* and *N. crassa* (Free, 2013)*.* The putative synthesis of β-1,6-glucan (moieties), however, may be increased as a result of the two Kre9/Knh1 like proteins found upregulated in the seven-fold *crh* mutant and encode homologues of KRE9 and KNH1 that known to synthesize β-1,6-glucan in *S. cerevisiae* (Brown and Bussey, 1993, Dijkgraaf et al., 1996). The increased presence of both CBM50 effector proteins—with putative chitinolytic protection and chitin tethering—combined with CwpA, PirA, AgtA and Kre9/Knh1 like proteins, may suggest an unknown cell wall stabilizing mechanism through which chitin release is prevented and additional protein to cell wall cross-linking occurs, resulting from the absence of Crh enzymes.

In line with minor changes in expression of some cell wall metabolic genes (Table 5, Table 6), we did not find evidence for differences in overall cell wall composition. A more detailed analysis of the fractionated cell wall using alkali fractionation only resulted in a small drop in both glucose and glucosamine in the AIF. However, no increase of glucose in either of the ASF-I or ASF-II fractions was observed, as previously reported upon a double deletion of *CRH1* and *CRH2* in *S. cerevisiae* (Rodríguez-Peña et al., 2000). Based on these data it is possible either that cross-links between chitin and glucan still exist or that other glucan linkages indirectly (e.g. β-1,4-glucan and β-1,6-glucan) connect to chitin and result in alkali insolubility. Recently, an *in situ* cell wall assessment of intact *A. fumigatus* cells*,* using solid state NMR, revealed the cell wall architecture and polymer interactions in great detail (Kang et al., 2018). Interestingly, they show presence of α-1,3-glucan in both the highly (alkali soluble) mobile outer shell and in the rigid hydrophobic core of the cell wall, tightly packed together with chitin. Their findings suggest that β-1,3/-1,6-glucans do not form the rigid backbone structure due to high levels of hydration and mobility, but instead enclose the rigid hydrophobic core that consists of chitin and α-1,3-glucan. Thus, chitin and α-1,3-glucan form the anchor to which β-glucans are proposed to be covalently attached to, while evidence for covalent interactions between α-1,3-glucan and β-glucan or chitin are still lacking. Nonetheless, these findings on intact cells shed new light on the way that we look at the filamentous fungal cell wall. Until now, no cell wall composition assessments have been performed to characterize the impact of removing the *crh* gene family in filamentous fungi. It is important to note that both species that have been studied for cell wall composition in relation to *CRH* loss, *S. cerevisiae* and *C. albicans,* do not possess cell wall α-1,3-glucans and have drastically lower relative amounts of cell wall chitin (Free, 2013). In a different yeast, *Schizosaccharomyces pombe,* vegetative cells neither possess cell wall chitin nor *CRH* enzymes, but do contain α-1,3-glucan which has been shown to be essential for growth (Grün et al., 2004, Hochstenbach et al., 1998). In filamentous fungi, absence of cell wall α-1,3-glucan has previously been reported to be non-lethal for *A. fumigatus, A. oryzae* and *A. nidulans* (Henry et al., 2012, Miyazawa et al., 2018, Miyazawa et al., 2016), as chitin may suffice for structural integrity. Presence of both chitin and α-1,3-glucan is unique for these filamentous fungi and may create an entirely different dynamic interplay of cell wall integrity. Therefore, based on the reported tight interaction between chitin and α-1,3-glucan and results from our studies, it is possible that loss of chitin to β-glucan cross-linking may not affect cell wall integrity of *A. niger* and *A. fumigatus* in the same way as in either of the aforementioned yeast studies.

Interestingly, we showed an important relation between α-1,3-glucan and chitin to β-glucan cross-linking for maintaining cell wall integrity. When exposed to CR, neither the seven-fold *crh* mutant nor the α-glucan synthase knockouts were negatively affected in growth, separately. However, when both *crh* and *ags* deletions were combined, a synthetic growth defect on CR was observed, showing the importance of Crh enzymes when α-glucan levels are reduced. Additionally, we showed that Crh enzymes are important when galactofuranose biosynthesis is abolished. A deletion of *ugmA* in the seven-fold *crh* family knockout resulted in heavily reduced growth a sporulation and increased sensitivity to CR. Previously reported expression data of the *ΔugmA* strain already revealed increased levels of *crhE* transcript, confirming the importance of Crh enzymes in maintaining cell wall integrity in absence of galactofuranose. In summary, these results suggest that Crh enzymes play an auxiliary role in maintaining cell wall integrity in the absence of other cell wall components.

Because α-1,3-glucan is lacking in cell walls of both *S. cerevisiae* and *C. albicans,* our findings are significant in explaining the discrepancy of the *A. niger* seven-fold *crh* mutant phenotype on CR with the literature on *CRH* knockouts in both *S. cerevisiae* and *C. albicans*. We propose that, in α-glucan-containing filamentous fungi, the chitin-α-1,3-glucan interaction and Crh enzymes may act as reciprocal backup systems to ensure cell wall integrity by anchoring chitin to the cell wall: Reduced α-glucan synthesis is remediated by Crh-facilitated cross-linking of chitin to β-glucans, whereas absence of chitin to β-glucan cross-linking in the seven-fold *crh* knockout strain may be compensated by the tight packing of chitin with α-1,3-glucan.

In contempt of the reported dispensable nature of Crh enzymes in *A. niger*, we are not able to rule out the existence of other cell wall chitin cross-links that may fortify the cell wall. However, we were able to show the importance of Crh enzymes in relation to both α-glucan and galactofuranose, assuming at least in-part loss of cell wall integrity by *crh* family disruption. For now, it remains difficult to explain why there are so many different *crh* genes in *A. niger* and other filamentous fungi. Especially if filamentous fungi possess an α-1,3-glucan-chitin-interaction redundancy mechanism that renders the *crh* gene family dispensable. Paradoxically, filamentous fungi possess more *crh* gene copies than yeast-like fungi that are devoid of cell wall α-glucan and such a putative redundancy mechanism. Despite this, α-glucan synthases most likely evolved post hyphal multicellularity, whereas the *crh*-facilitated β-glucan to chitin transglycosylation coincided with the evolution of hyphae (Kiss et al., 2019). As such, this chronological order of development would allow selection pressure to drive the diversification of Crh enzymes during different growth phases of filamentous fungi, prior to the introduction of cell wall α-glucan. Apart from its role in pathogenesis and conidial aggregation (Yoshimi et al., 2017), α-glucan may thus have the added benefit of aiding chitin-to-cell-wall anchorage—next to Crh facilitated chitin-β-glucan cross-links—in order to fortify cell wall structure.

## 5 Conclusions

Fungi show a high level of complexity with various mechanisms of redundancy to ensure integrity of the cell wall. Not just on the level of multiple gene copies in a single family, but also in terms of inter-connected architectural layers within the macrostructure of the cell wall itself. The complete removal of the *crh* gene family affected neither growth rate nor did it increase the sensitivity towards cell wall stressing compounds, suggesting dispensability of Crh enzymes for cell wall integrity. However, we showed that removal of other cell wall components—through interference of either α-glucan or galactofuranose synthesis—increased susceptibility to cell wall stressing compounds in the seven-fold *crh* deletion mutant compared to the same disruptions in the wild type. Reduced levels of either α-glucans or galactofuranose may increase the cell wall’s dependence on chitin for integrity which, in turn, may require Crh facilitated crosslinks to warrant cell wall stability. Taken together, these findings suggest that inter-structural mechanisms of redundancy are present in filamentous fungi, and only just scratch the surface in understanding cell wall complexity. With special regard to identification of new anti-fungal compounds and strategizing anti-fungal treatments, understanding these mechanisms beckons the need to continue cell wall research in filamentous fungi.

## CRediT authorship contribution statement

**Tim M. van Leeuwe:** Conceptualization, Investigation, Validation, Formal analysis, Methodology, Writing - original draft, Visualization. **Jasper Wattjes:** Investigation, Validation, Formal analysis, Visualization. **Anna Niehues:** Investigation, Validation, Formal analysis, Visualization. **Gabriel Forn-Cuní:** Software, Formal analysis, Visualization. **Nicholas Geoffrion:** Software, Resources. **Hugo Mélida:** Investigation, Validation. **Mark Arentshorst:** Investigation. **Antonio Molina:** Funding acquisition. **Adrian Tsang:** Resources. **Annemarie H. Meijer:** Resources. **Bruno M. Moerschbacher:** Funding acquisition. **Peter J. Punt:** Conceptualization, Writing - review & editing, Funding acquisition. **Arthur F.J. Ram:** Conceptualization, Writing - review & editing, Funding acquisition, Data curation, Supervision.

## Declaration of Competing Interest

The authors declare that they have no known competing financial interests or personal relationships that could have appeared to influence the work reported in this paper.

## Appendix A Supplementary data

Supplementary Table S1 Expression data of *crh* genes during spore maturation at 2 days, 5 days and 8 days. Data from Teertstra et al., 2017.

Supplementary Table S2 Expression data of differentially expressed genes. Includes highlighted cell wall integrity (CWI) genes and cell wall biosynthetic genes as described by Pel et al., 2007.

Supplementary Table S3 Gene Ontology (GO) analysis.

Supplementary Table S4 Yield percentages (dry weight/starting dry weight) after alkali fractionation and dialysis from individual bioreactor samples (BR_TvL_#). Biological replicate cell wall samples from both wild type (MA234.1; BR_TvL_15 and BR_TvL_18) and seven-fold crh mutant (TLF39; BR_TvL_16 and BR_TvL_18) were fractionated as described in Section 2.4.1.
